# Genetic design of enhanced valley splitting towards a spin qubit in silicon

**DOI:** 10.1038/ncomms3396

**Published:** 2013-09-09

**Authors:** Lijun Zhang, Jun-Wei Luo, Andre Saraiva, Belita Koiller, Alex Zunger

**Affiliations:** 1 University of Colorado, Boulder, Colorado 80309, USA; 2 National Renewable Energy Laboratory, Golden 80401, Colorado, USA; 3 Instituto de Fisica, Universidade Federal do Rio de Janeiro, Caixa Postal 68528, 21941-972 Rio de Janeiro, Brazil

## Abstract

The long spin coherence time and microelectronics compatibility of Si makes it an attractive material for realizing solid-state qubits. Unfortunately, the orbital (valley) degeneracy of the conduction band of bulk Si makes it difficult to isolate individual two-level spin-1/2 states, limiting their development. This degeneracy is lifted within Si quantum wells clad between Ge-Si alloy barrier layers, but the magnitude of the valley splittings achieved so far is small—of the order of 1 meV or less—degrading the fidelity of information stored within such a qubit. Here we combine an atomistic pseudopotential theory with a genetic search algorithm to optimize the structure of layered-Ge/Si-clad Si quantum wells to improve this splitting. We identify an optimal sequence of multiple Ge/Si barrier layers that more effectively isolates the electron ground state of a Si quantum well and increases the valley splitting by an order of magnitude, to ∼9 meV.

## Abstract (web summary)

Electronic spins in Si are potentially useful in the development of solid-state quantum devices, but its degenerate valley states limits this potential. Zhang *et al.* use a genetic algorithm to identify a Ge/Si-multilayer-clad Si quantum structure whose valley splitting is increased by an order of magnitude.

## Graphical abstract

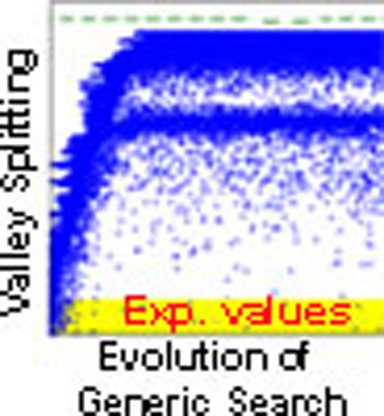

The qubits for quantum information processing are encoded in two-level quantum systems {|0〉, |1〉} (ref. 1), and can be realized, for example, by two spin states {|↑〉, |↓〉} of an electron at the conduction band edge of a semiconductor^2^,^3^,^4^. Although Si enjoys a number of advantages over III–V semiconductors in this respect, including long spin coherence lifetime (associated with its weak spin–orbit coupling and small content of non-zero nuclear-spin isotopes)^5^,^6^, as well as advanced fabrication know-how, its major drawback is the (sixfold) orbital degeneracy of its lowest conduction band (Fig. 1a) located close to the *X* point in the Brillouin zone. This is no longer a two-level system determined solely by its spin, leading to considerable leakage and decoherence driven by the energetic proximity among the degenerate orbitals^6^,^7^. Whereas this six-fold valley degeneracy in the O_*h*_-symmetric bulk Si can be partially removed by application of tensile biaxial strain^8^, thus, isolating the two lowest |+*z*〉 and |−*z*〉 components from the rest (Fig. 1b), the creation of a sufficiently large energy splitting within this *Z*-valley subspace (hereby called valley splitting (VS), see Fig. 1c) has proven to be a challenge for the experimental realization of spin-only qubits in Si^6^. This is clearly indicated by the very limited range of VS (of the order of 1 meV or less) attainable for Si quantum wells surrounded by Ge–Si alloy barriers in experiment^9^,^10^,^11^,^12^,^13^,^14^,^15^ and theory^16^,^17^,^18^,^19^,^20^,^21^,^22^,^23^, which seriously hinders the further development of Si-based quantum computation.

The geometry of the basic physical system explored (Fig. 1d) includes a Si slab (well) interfaced by a material with higher conduction band (barrier). The VS of this system depends on a multitude of degrees of freedom present in the actual device growth. The Si well of thickness *d* cladded by barrier materials of composition *X*_b_ is coherently strained on a substrate with the planar lattice constant *a*_s_ (determined by its composition *X*_s_). We focus on the substrate and barrier composed of Ge–Si-based materials, which provide better-quality interfaces than oxides. The barrier can be a Ge–Si random alloy of composition *X*_b_ or any corresponding atomistically ordered structure. We incorporate monolithically the full system containing up to 10^5^ atoms per computational cell, via an atomistic pseudopotential Hamiltonian^24^,^25^, solved in a plane-wave basis for each relaxed atomic configuration, which gives directly the energies {*ɛ*_*i*_} and wave-functions {Ψ_*i*_} of the valley states.

Focusing on this basic system, here we demonstrate in a systemical way how atomically resolved degrees of freedom in the Si well, barrier materials and substrate could raise the VS. We found that although Ge–Si disordered random alloy barriers always give a limited value of VS (below 1 meV), which is consistent with previous theoretical and experimental results^9^,^10^,^11^,^12^,^13^,^14^,^15^,^16^,^17^,^18^,^19^,^20^,^21^,^22^,^23^, the atomic-scale ordering in Ge–Si layer-by-layer superlattice-like barriers can be effectively engineered to tune the VS over a wide energy range. This finding, combined with the efficient selection capabilities of a biologically inspired genetic algorithm search^26^,^27^, allows us to identify the superlattice barriers with the optimum Ge/Si layering sequence, providing one order of magnitude enhancement of the VS with respect to alloy barriers. The enhanced VS is robust under reasonable inter-layer mixing between different species, and is interestingly ‘protected' even if some larger mixing occurs.

## Results

### Macroscopic degrees of freedom

**Figure 1:**
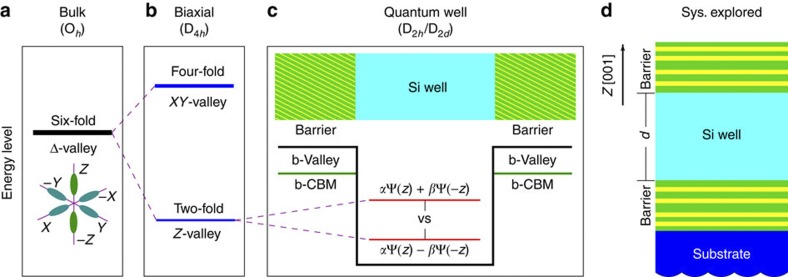
Description of valley splitting and system explored. Schematic representation of (**a**) the (sixfold degenerate) Δ-valley state of bulk Si; (**b**) splitting between *XY*-valley and *Z*-valley by tensile biaxial strain; and (**c**) splitting of *Z*-valleys by the abrupt interfaces of the quantum well. Note that the confinement barrier height for the well is the band offset (between Si well and barrier) of *Z*-valley (b-Valley), higher than the band offset of conduction band minima (b-CBM). The symmetry of Si quantum well of *N* monolayers alternates from D_2*d*_ ↔ D_2*h*_ for *N* odd ↔ even, respectively. (**d**) Heterostructure geometry adopted in the present study. A [001]-oriented Si quantum well of thickness *d* and the surrounding barriers are coherently strained by epitaxial growth over a specified substrate. Both barrier and substrate are composed of Si–Ge-based materials.

**Figure 2:**
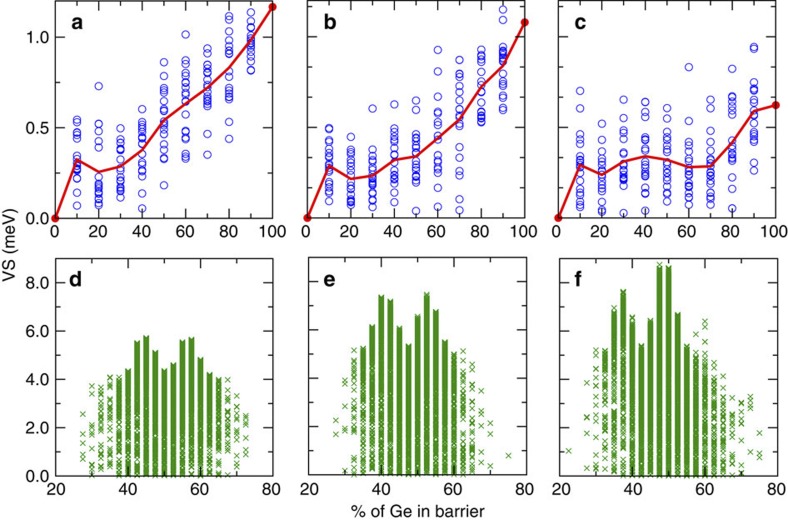
Si well embedded in disordered and ordered Si–Ge barriers. Calculated VS (in meV) as a function of the content of Ge in the barrier, for a 40 MLs Si quantum well embedded in Si–Ge disordered alloy (**a**–**c**) and ordered superlattice (**d**–**f**) barriers, on a 0, 20 and 40% Ge substrate. Solid red lines in (**a**–**c**) represent the configuration-averaged VS for alloy barriers and empty circles represent specific atomic realizations (20 for each composition). In **d**–**f**, the superlattice barrier has a 80 MLs thickness, and structural configurations (green crosses) are generated by the genetic algorithm search (see Fig. 4). Note that the VS for superlattice barriers (**d**–**f**) is given in an energy scale (*y* axis) with ∼1 order of magnitude larger than that of alloy barriers (**a**–**c**).

We start by exploring the continuum configuration-averaged degrees of freedom in this system, as common in the literature^16^,^17^,^18^,^19^,^21^,^28^, finding that although they do not provide a clear avenue to major VS enhancement, their exploration hints at the importance of another length scale. We consider a fixed-thickness Si well embedded in the Ge–Si alloy barriers with varied composition *X*_b_, on three substrates with different composition *X*_s_. For each alloy composition *X*_b_ of barriers, we calculated 20 randomly realized atomic configurations and the averaged VS is evaluated. The solid red line in Fig. 2a–c shows the calculated configuration-averaged VS as a function of composition *X*_b_. Generally, one observes an uneventful monotonic increase of the averaged VS as the barrier becomes richer in Ge (see also Supplementary Fig. S1b, which shows the VS for a few distinct *X*_b_). Such continuum-like effect of the configuration-averaged alloy barriers can be understood by the gradual change of the barrier height. That is, the band offset between the valley states of Si well and barrier (the b-Valley term in Fig. 1c and Supplementary Fig. S1a)^21^,^23^. Although the averaged VS (red lines in Fig. 2a–c) shows substantial dependence on the epitaxial strain (also see Supplementary Fig. S1b), the variation of the macroscopic barrier composition *X*_b_ and substrate composition *X*_s_ provides limited tuning of VS.

### Atomically resolved length scale

Important clues emerge as to the significance of the atomically resolved length scale and symmetry, as indicated in a recent work on the intervalley splittings of PbSe^29^. In principle, the splitting within the *Z*-valleys is closely related to the interface-induced deviation from the O_*h*_ symmetry of bulk Si (or the D_4*h*_ symmetry of biaxially strained Si). For a Si quantum well (Fig. 1d), the interfacial perturbation potential Δ*V* with the D_2*h*_/D_2*d*_ symmetry provides a coupling channel between two *Z*-valley states, giving a VS magnitude in perturbation theory of 2|〈+*z*|Δ*V*|−*z*〉|. To tune VS, we can engineer the magnitude and profile of the perturbation potential Δ*V* by varying the atomic-scale structure and symmetry for the well and barriers. The importance of the atomic scale is revealed, for example, in Fig. 2a–c, where the blue circles represent the VS obtained by resolving distinct random realizations of site occupations in alloy barriers. The VS ranging from zero to an upper bound of ∼1 meV is in reasonable agreement with experiments^9^,^10^,^11^,^12^,^13^,^14^,^15^. We can see that the VS of Si can vary significantly for different atomic configurations of barriers at the same composition *X*_b_. This is consistent with the recent calculation showing that specific atomic arrangements at the interface region can result in distinct VS (however the assumed Si_3_Ge luzonite structure is difficult for experimental realization)^30^. Also, the critical role of atomic resolution and symmetry is apparent by considering a system of short-period Si–Ge superlattices located directly on a substrate (that is, no active Si layer in Fig. 1d), where our calculated VS reaches values as large as several tens of meV, although the Si–Ge superlattice system is not the case of interest here (but may relate to different qubit proposals^4^).

Inspired by these basic insights from the atomic length scale, we next explore in a systematic way whether and how atomic degrees of freedom in the Si well, in the composition and structure of the barrier, and in the epitaxial substrate could raise the VS. By varying the above degrees of freedom, we aim at identifying how the relevant physical factors affect VS and use it to seek an optimized combination maximizing the VS.

### Effect of Si well thickness

**Figure 3:**
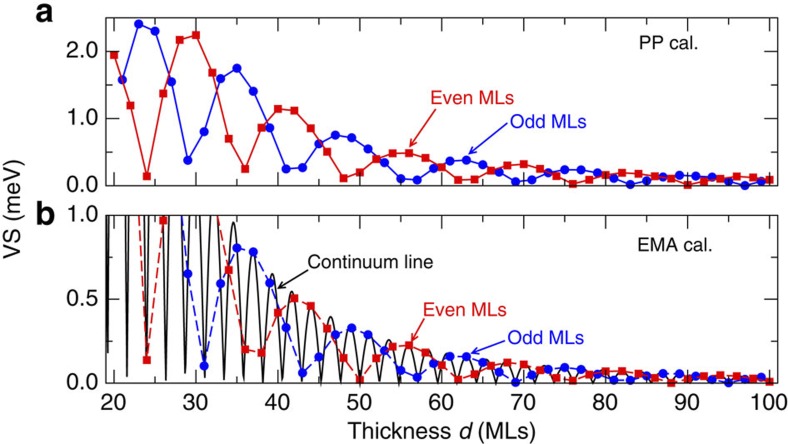
Dependence of valley splitting on Si well thickness. Calculated VS (in meV) as a function of the thickness *d* (in MLs) of a Si quantum well embedded in fixed pure Ge barrier on pure Si substrate. The pseudopotential (PP) and effective mass approximation (EMA) results are shown in (**a**) and (**b**), and the data at the odd and even number of MLs are shown in blue and red, respectively. For comparison with PP data, we show the EMA results as a continuum line with markings at the integer MLs. Note that the EMA results are smaller in magnitude than the PP results.

The thickness *d* of the Si well is the first obvious parameter to tune the perturbation potential Δ*V*, and thus manipulate VS. Figure 3a shows the dependence of VS on the thickness *d* in monolayers (MLs, 1 ML is equal to 1/4*a*_0,_ where *a*_0_ is the conventional cubic lattice constant) for fixed pure Ge barrier on pure Si substrate from the pseudopotential calculations. We observe an overall decay in the magnitude of VS as the thickness *d* increases, whereas the VS for *d* with an odd (blue circles) and even (red squares) number of MLs seems to oscillate independently, with a common period ∼14 ML and a phase shift of *π*/2. This intriguing oscillatory behaviour has been reported previously, and was attributed to the symmetry change of the Si well with *d* MLs: D_2*d*_↔D_2*h*_ for *d* odd ↔ even^16^,^17^,^31^. In Fig. 3b, we show the calculated VS within the effective mass approximation (EMA) as a function of the continuum thickness *d*_con_ (solid black line), as well as the data sampled at odd (blue circles) and even (red squares) atomic MLs. We find that although the EMA results with continuum *d*_con_ show a much faster oscillation, clearly they reproduce well the existence of independent oscillation for discrete *d* of odd and even MLs. Thus, we attribute this atomic-scale odd–even independent oscillation to a manifestation of the aliasing effect (introduced by sampling a function at a rate which is not fine enough to capture each oscillation), rather than to a symmetry change (see Supplementary Note 1 for detailed description). This understanding underlines that to gain an optimized VS of Si well, a well-controlled growth of monolayer precision is required to reach the thickness *d* at the peak of the oscillation.

### Atomically ordered superlattice barriers

**Figure 4:**
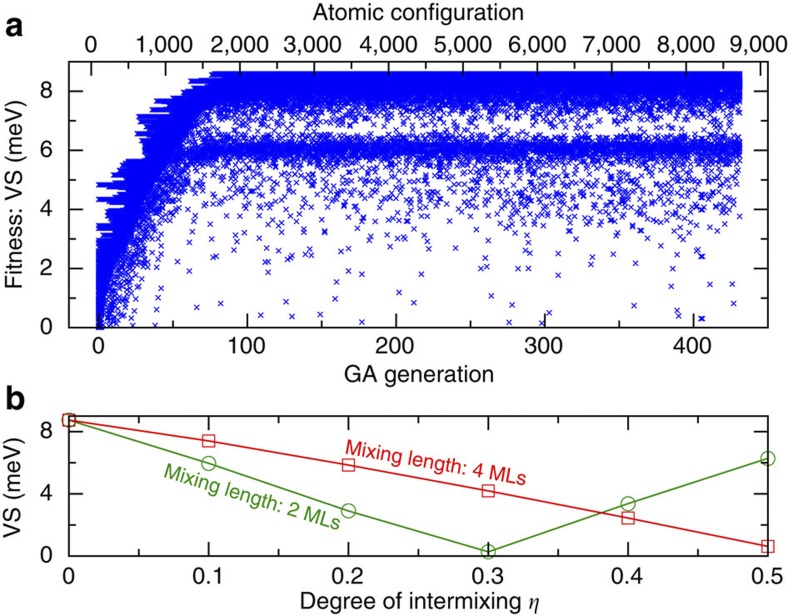
Genetically optimized valley splitting and inter-layer mixing. (**a**) Evolution of fitness (VS) with generation in an inverse-band-structure calculation for a 40 MLs Si quantum well embedded in ordered superlattice barriers with the 80 MLs thickness, on a 40% Ge substrate. The top axis shows the number of atomic configurations investigated during the evolution. (**b**) Calculated VS (in meV) for the optimum configuration of superlattice barrier achieved in **a**—Ge_4_Si_6_Ge_2_Si_6_Ge_4_Si_4_Ge_4_Si_4_…, as a function of the degree of inter-layer mixing, *η* (see text). At *η*=0 there is no mixing and *η*=0.5 means the maximum diffusion, that is, complete destruction of Si-rich or Ge-rich pattern layer. We explore two cases of mixing length: 1 ML for each side of the interface—total of 2 ML (green); and 2 ML for each side—total of 4 ML (red), defining the maximum range at the interface where the mixing occurs. For each *η*, 10 alloy realizations are randomly sampled and the averaged VS is shown as a line. The fluctuation induced by different alloy realizations is within 0.2 meV.

The substantial effect of specific atomic realization for the disordered alloy barriers (open circles in Fig. 2a–c) stimulates us to investigate the situation where the barriers are composed of ordered superlattices, that is, a repeated sequence of Si and Ge layers of arbitrarily assigned widths. We explore the film (as shown in Fig. 1d) composed of a 40 MLs Si well inserted between two superlattice barriers located symmetrically on each side of Si well and each having a thickness of 80 MLs. The film is to be grown on the substrates with the Ge contents of 0, 20 and 40%. Note the even (40) MLs Si well corresponds to the even peak in Fig. 3a. The minimum stacking unit of each Si/Ge species is chosen as the bilayer to simulate realistic growth constrains, as demonstrated in the molecular beam epitaxy approach^32^ (if this constrain can be overcome, the configuration space is richer and even higher VS is probably achievable). This gives an astronomical number (2^40^) of candidate layer-stacking configurations of barriers, so a direct calculation for enumeration of all the candidates is not practical. We perform an inverse-band-structure search calculation^26^,^27^ where the best fitness is defined by the maximum VS and favourable structures are selected within a genetic algorithm approach. This method holds a unique advantage in optimizing any desired target electronic property in the configuration space showing a combinatorial burst of degrees of freedom, pinpointing the global optimum structure^27^,^33^,^34^,^35^,^36^. Figure 4a shows the evolution of fitness (VS) with generation (evolution step). One clearly observes that the VS can be effectively tuned within a wide energy range, from negligibly small up to ∼9 meV, by varying the Ge/Si stacking sequence of superlattice barriers. Less than 100 generations already identify the best individuals, which remain superior for the following hundreds of generations, while new individuals still emerge with intermediate VS values.

Figure 2d–f shows the achieved VS of all the atomic configurations visited by the inverse-band-structure search, sorted in terms of the Ge content in the barriers on three varied substrates. It is demonstrated that a remarkable VS enhancement by a factor of 5–10 is achievable with ordered superlattice barriers as compared with disordered alloy barriers (Fig. 2a–c) for all substrates. Table 1 lists the explicit value of maximum VS and the corresponding optimum configuration of barrier. Comparing with the maximum VS for the disordered alloy barriers—∼1.0 meV on all the substrates, the maximum VS for the ordered superlattice barriers reaches 5.7, 7.4 and 8.7 meV on the 0%, 20% and 40% Ge substrate, respectively. From Fig. 2d–f, we also find that the multilayer superlattice barriers show larger VS around the central region, at 40–60% Ge content in the barrier, different from higher Ge content leading to larger VS for random alloy barriers. The same Ge content in the superlattice barriers can lead to both high and low VS extremes, again emphasizing the key role of atomistic scale ordering in controlling VS. Note that a high epitaxial strain (produced by a large Ge content in the substrate relative to the Ge-poor film to be grown) is not an essential condition for significant enhancement of VS. This is clearly reflected by the fact that the optimum configuration with no Ge in the substrate gives a VS magnitude of 5.7 meV and that the optimal configuration with the 20% Ge substrate shows a VS of 7.4 meV (Table 1). Although these values are somehow lower than that of the optimum configuration on the 40% Ge substrate (8.7 meV), the enhancement is still remarkable by comparison with the values for alloy barriers. As, nonetheless, our film (as in Fig. 1d) is coherently strained on the prescribed substrate, we have analysed the effect of possible dislocation formations (that are widely observed in strained epitaxial film growths). Analysis of the critical thickness for the growth of Si well surrounded by the above optimum configurations of barrier indicates that experimental realization in a dislocation-free growth mode is rather promising. Even if a moderate dislocation density was to appear in the thick-layer growth, the effect of their induced elastic strain field on the VS magnitude is expected to be rather modest (see Supplementary Note 2 for more information).

### The Si/Ge_4_ motif

**Figure 5:**
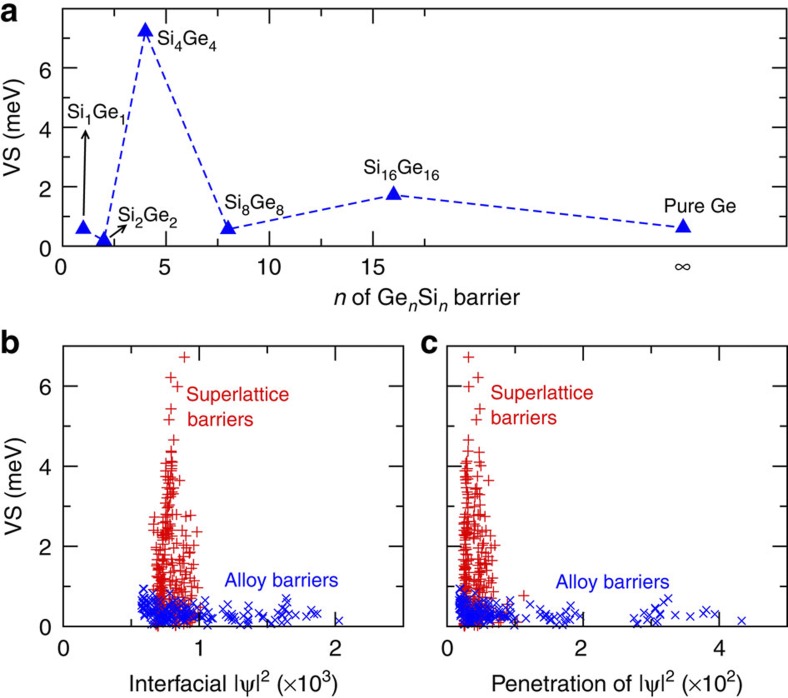
Ge_*n*_Si_*n*_ barriers and insights from wave-functions. (**a**) Calculated VS (in meV) for a 40 MLs Si quantum well embedded in the Ge_*n*_Si_*n*_ (*n*=1, 2, 4, 8, 16) superlattice barriers on a 40% Ge substrate. The result for pure Ge barrier (*n*=∞) is shown for comparison. (**b**,**c**) Calculated VS (in meV) shown as a function of (**b**) planar-averaged (in the *XY* plane) wave-function squared magnitude at the interface (
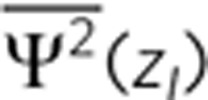
), and (**c**) planar-averaged wave-function squared penetration into the barriers 
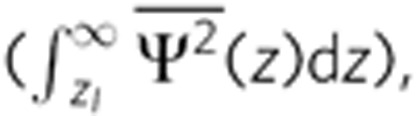
 for a 40 MLs Si quantum well embedded in random alloy and 16-ML period (see Supplementary Fig. S2 for detailed description) superlattice barriers, on a 40% Ge substrate. The averaged wave-function magnitude between two *Z*-valley states is used for such calculations.

Interestingly, all the optimum configurations identified start the barrier sequence by a Ge_4_ sublayer (see Table 1). This same magic thickness for the first Ge sublayer is also identified in the exhaustive enumeration calculations for the superlattice barriers with a shorter period of 16 MLs (see Supplementary Fig. S2a–c). Similar results are obtained for an Si well with the thickness of 47 MLs (located at an odd peak of Fig. 3a, see Supplementary Fig. S2d–f). To better understand this, we explore a simple case—the fixed 40 MLs Si well embedded in Ge_*n*_Si_*n*_ superlattice barriers with *n*=1, 2, 4, 8, 16, as shown in Fig. 5a. We see that the barrier of Ge_4_Si_4_ superlattice indeed exhibits the largest VS (>7 meV), whereas all other barriers (including pure Ge) show typically low VS (<2 meV). This indicates that the starting sublayer thinner or thicker than Ge_4_ seems to equally suppress VS. We unravel the underlying origin within the EMA context. Briefly, the VS induced by an Si/Ge (ascending offset) interface has opposite sign to the Ge/Si (descending offset) interface with the same wave-function. Choosing the interface positions to match the maxima/minima of the VS at the ascending/descending interfaces would maximize the total VS. It is impossible to match the interface positions perfectly to the incommensurate oscillations of well-thickness-dependent VS (Fig. 3), but the Ge_4_ sublayer is the closest we can get to this matching within the bilayer growth constraint we impose (better commensurability would be achieved if we chose to analyse any layer thickness, including odd numbers of MLs). Conversely, starting with a Ge_2_ sublayer cladding the Si well, we find a destructive interference, in agreement with the suppressed VS for Ge_2_Si_2_ superlattice barrier in Fig. 5a. This engineering is analogous to that of a distributed Bragg reflector (see Supplementary Note 3 for detailed description). But the fact that the oscillations are incommensurate with the lattice and the strong dependence of VS on atomic ordering makes it impossible to analytically predict the optimal structure. For this reason, the genetic selection of candidate structures is an essential ingredient of this work.

Previous studies correlate the VS with the electronic wave-function magnitude of the *Z*-valley at the interface (interfacial |Ψ|^2^)^17^,^19^,^21^,^23^ and the wave-function penetration into the barrier region (penetration of |Ψ|^2^)^21^,^23^. In Fig. 5b,c, we probed the VS as a function of interfacial |Ψ|^2^ and penetration of |Ψ|^2^, for the 40 MLs Si well cladded by alloys barriers (blue crosses) and superlattice barriers (red pluses). Compared with the alloy barriers, the stronger confinement power of superlattice barriers give much narrower distribution of both interfacial |Ψ|^2^ and penetration of |Ψ|^2^. The optimum VS values for the superlattice barriers emerge in the region of the narrowest distribution of these two quantities. This is related to the sharp well/barrier interface for superlattice barriers, which could in principle enhance the VS^21^,^37^.

### Effect of Ge–Si intermixing in barriers

As it is still a challenge to grow a perfectly pure sublayer of Si or Ge in superlattices due to atomic inter-diffusion^38^, we examine how much VS is affected by the interfacial mixing between Si and Ge. In particular, the inter-layer mixing is modelled by mapping pure Si into Si_1−*η*_Ge_*η*_ and pure Ge into Ge_1−*η*_Si_*η*_ at the interfacial first few layers, determined by a mixing length. The parameter *η* quantifies the degree of inter-layer mixing, with *η*=0 corresponding to no mixing and *η*=0.5 meaning maximum mixing, that is, complete destruction of Si-rich or Ge-rich pattern within this layer. Figure 4b shows the calculated VS as a function of *η* for the above optimized superlattice barrier on %40 Ge substrate (Ge_4_Si_6_Ge_2_Si_6_Ge_4_Si_4_Ge_4_Si_4_…, see Supplementary Fig. S3 for more ordered superlattice barriers), when two cases of mixing lengths (two MLs (green) and four MLs (red)) are explored. Note that the favourable Ge_4_ starting sublayer is only partially damaged if the mixing length is 2 ML (1 ML towards each side of the interface), whereas for the 4 ML mixing length the Ge-pure layer is totally destroyed. The non-trivial, non-monotonic behaviour indicates that the intermixing may lead to the formation of a more complex geometry which tunes VS by affecting the interference pattern discussed before. This is reflected in a surprisingly steeper suppression of VS in the shorter mixing length of two MLs compared with the longer mixing length of four MLs for small *η*. Also, at very large *η*, the structure becomes a complex layering of alloys, pure Si and pure Ge, which might keep suppressing (the case of 4 ML) or inverting the symmetry and enhancing the VS (the case of 2 ML). In both mixing lengths, for a reasonable degree of mixing (*η*<0.1), the rather high VS of >6 meV is preserved.

## Discussion

We anticipate that the choice of ordered superlattice barriers instead of random alloy barriers might mitigate many problems of real samples. For instance, the intrinsic non-deterministic nature of alloys induces disorder ranging from the geometry of the interface plane to the inhomogeneous strain fields^39^. The leakage of electrons tunnelling through the superlattice barrier should also be suppressed as the electronic density inside the barrier is much reduced. The structure we proposed is accessible within the current experimental fabrication capabilities such as the molecular beam epitaxy^8^,^38^,^40^,^41^ and chemical vapour deposition^8^,^40^,^41^,^42^,^43^,^44^. Particularly in the latter approach, the hydrogen surfactant effect, which can reduce the surface segregation of Ge, can facilitate production of the Si/Ge heterostructures with more abrupt Ge profiles^8^,^41^. The identification of a simple motif of Si and Ge layer sequence of multilayer barriers that sustains large valley splitting can overcome, in a practical systematic way, the long standing issue on the degraded fidelity of spin-only qubits in Si, thus providing a roadmap for reliable Si-based quantum computing. This work also implies that the task of finding the optimal architecture for quantum computation is often a combinatorial materials science challenge and could be addressed via modern theory of material-by-design.

## Methods

### Structures for VS optimization

The structures employed to optimize VS in this work (Fig. 1d) involve an active Si well with the thickness of *d* MLs, cladded on both sides by a barrier consisting of Si–Ge-based materials, including homogeneous random alloy and layer-by-layer superlattice structures. The whole system is coherently strained on a substrate, via minimization of atomically resolved strain with a generalized valence force field method (see Supplementary Methods)^45^. To comply with what is currently accessible in experimental (for example, molecular beam epitaxy and chemical vapour deposition) growth^8^,^41^ we used two restrictions: (i) as too high Ge content in substrate is known to cause dislocations in thick Si active layers to relieve excessive strain, the Ge content in the substrate is limited up to 40%. (ii) a bilayer is used as the minimum stacking unit of each species (Si/Ge) for the superlattice barrier^32^.

### Atomistic empirical pseudopotential calculations

The energies and wave-functions of conduction valley states for candidate structures are calculated ‘on the fly' with the atomistic pseudopotential method, described in detail in Zunger^24^ and Wang and Zunger^25^. The atomistic pseudopotential method (overcoming the well-known Density-Functional-Theory limitations on electronic structure calculations), accompanied with a plane-wave basis set and folded-spectrum diagonalization, allow us to accurately calculate energy splitting of *Z*-valley states (at the order of meV or lower) for numerous candidate structures with economic efficiency, as described in Supplementary Methods.

### Effective mass calculations

Effective mass calculations were performed to accompany the interpretation of the pseudopotential results. The effective mass calculations follow essentially the model presented in Saraiva *et al.*^21^,^23^ and Saraiva *et al.*^23^, adapted to describe quantum wells in first order approximation, as described briefly in Supplementary Methods.

### Inverse-band-structure approach

For Si wells embedded in layer-by-layer superlattice barriers, as the search space shows a combinatorial burst of degrees of freedom, we employ the developed inverse-band-structure approach^26^,^27^,^35^,^36^, a biologically inspired (Darwinian) genetic algorithm to guide the electronic structure calculations, with the aim at finding the optimum configuration that gives the maximum VS (Supplementary Methods).

## Author contributions

A.Z. and B.K. conceived the project. L.Z. carried out the atomistic pseudopotential calculations and adapted the genetic algorithm search. A.S. carried out the effective mass calculations. L.Z., J.-W.L., A.S., B.K. and A.Z. contributed to the design of the project, the analysis of results and the writing of the paper.

## Additional information

**How to cite this article:** Zhang, L. *et al.* Genetic design of enhanced valley splitting towards a spin qubit in silicon. *Nat. Commun.* 4:2396 doi: 10.1038/ncomms3396 (2013).

## Supplementary Material

Supplementary Information Supplementary Figures S1-S3, Supplementary Notes 1-3, Supplementary Methods and Supplementary References

## Figures and Tables

**Table 1:** Optimal valley splitting achieved with superlattice barriers.

**Substrate**	**Maximum VS (meV)**	**Optimum configuration of barrier**
%0 Ge	5.7 meV	Ge_4_Si_4_Ge_2_Si_6_Ge_4_Si_4_Ge_4_Si_2_…
%20 Ge	7.4 meV	Ge_4_Si_4_Ge_4_Si_2_Ge_4_Si_6_Ge_4_Si_2_…
%40 Ge	8.7 meV	Ge_4_Si_6_Ge_2_Si_6_Ge_4_Si_4_Ge_4_Si_4_…

The maximum VS and corresponding optimum configuration of ordered superlattice barrier identified by the inverse-band-structure search calculations (as shown in Fig. 2d–f). The Si well thickness is fixed to 40 MLs and the content of Ge in substrate ranges from 0 to 40%. The optimum configuration of barriers is given in the sequence of Si/Ge monolayers counted from the well boundary. Note the favorable Ge_4_ starting sublayer in all cases.
