# Supplementary material for: Genetic design of enhanced valley splitting towards a spin qubit in silicon
Source: Nat Commun. 2013 Sep 9;4:2396. doi: 10.1038/ncomms3396 (PMC3778719; doi:10.1038/ncomms3396)
Supplement: Supplementary Information — Supplementary Figures S1-S3, Supplementary Notes 1-3, Supplementary Methods and Supplementary References [file ncomms3396-s1.pdf]

Supplementary Figures

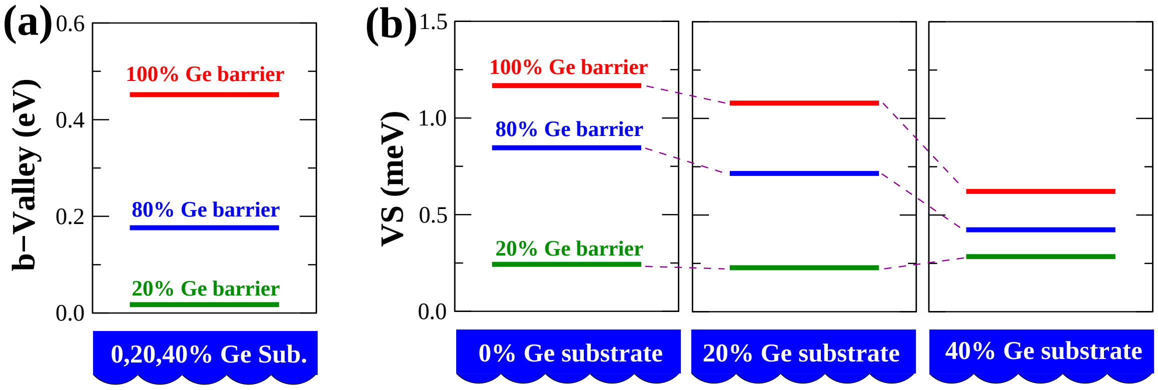

Supplementary Figure S1: **Configuration-averaged barrier height and VS.** (a) Calculated barrier height (b-Valley, in eV) and (b) VS (in meV) for a 40 MLs Si quantum well embedded in the indicated Ge-Si random alloy barriers on different substrates. Three barriers are considered: Si-rich (20% Ge barrier), Ge-rich (80% Ge barrier), and pure Ge (100% Ge barrier). For the Si-rich and Ge-rich barrier, the configuration-averaged barrier height and VS are shown among 50 randomly selected atomic configurations of alloy barriers. We find negligible change of b-Valley for different epitaxial strain under three substrates, as shown in (a).

The barrier height is usually a well-defined physical quantity governing VS of Si in the continuum-like approach such as effective mass calculations. We explore the effect of barrier height by changing the Ge content in alloy barriers, and substrate strain by considering different substrates. Note that the barrier height corresponds to the band offset between the valley states (b-Valley) of Si well and barrier, rather than the band offset between conduction band minima (b-CBM), as depicted in Fig. 1c of the main text. Supplementary Figure S1a gives the configuration-averaged barrier height of Ge_xSi_{1-x} alloy barriers ($x = 1, 0.8$ and 0.2). As expected, the barrier height increases with the Ge content in barriers, but is insensitive to substrate-induced epitaxial strain. The calculated configuration-averaged VS for a 40 MLs well cladded by above alloy barriers on different substrates is given in Supplementary Figure S1b. For each substrate, the VS increases with barrier composition from Si-rich ($x = 0.2$), to Ge-rich ($x = 0.8$), to pure Ge ($x = 1$). The barrier height and the VS value follow, both in a non-linear dependence, the same trend with the Ge content. The VS values are clearly sensitive to epitaxial strain, which indicates that strain distorts the electron wave-function in the Si well, particularly around the interface region — which mostly affects the VS [17, 19, 21].

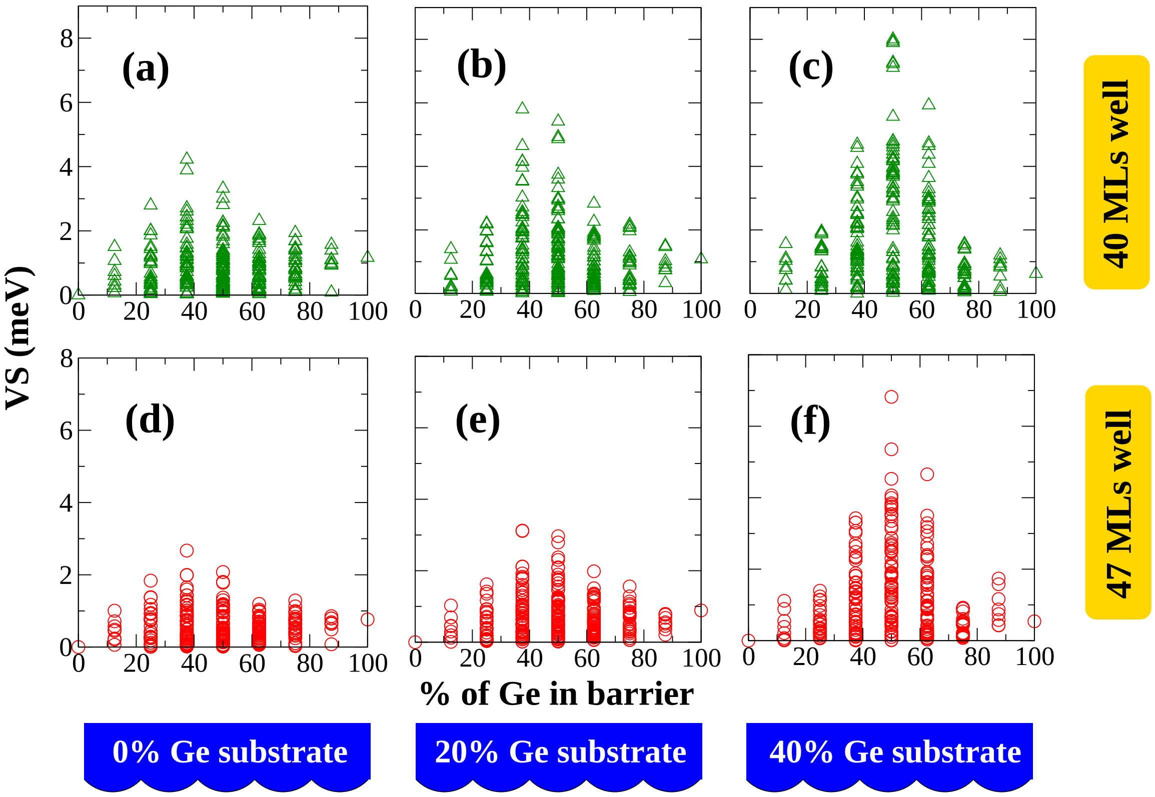

Supplementary Figure S2: **Cases of superlattice barriers with the shorter period.** Calculated VS (in meV) as a function of the content of Ge in the barrier, for a 40 MLs (green triangles, a, b, c) and 47 MLs (red circles, d, e, f) Si quantum well embedded in ordered superlattice barriers, on a 0%, 20%, and 40% Ge substrate. The superlattice barrier has a 16 MLs period, where each bilayer is composed of either Si or Ge, leading to 2^8 possible structural configurations.

Complementary to the inverse-band-structure search for the superlattice barrier with a total thickness of 80 MLs on each side of Si well, we explore the case of Si well surrounded by the superlattice barrier with a shorter period of 16 MLs (where the total thickness is still 80 MLs, containing five periods). Still with the minimum stacking unit of bilayer, this gives 2^8 possible configurations, which can be completely captured by the direct enumeration calculations. Complementary to the well thickness of 40 MLs (located at an even peak of Fig. 3a in the main text) chosen in the main text, we consider another well thickness of 47 MLs (located at an odd peak of Fig. 3a in the main text). Supplementary Figure S2 shows the calculated VS of all configurations for 40 MLs (a,b,c) and 47 MLs (d,e,f) Si well, sorted in terms of the Ge content in the barriers on three varied substrates. Clearly, these results from direct calculations in the smaller configuration space have implied some main features of the results from inverse-band-structure search in Fig. 2d-f of the main text. Particularly, for both two well thicknesses, the superlattice barriers

exhibit larger VS at 40-60% Ge content in the barrier. The same Ge content in the barriers leads to both high and low VS extremes. On all the substrates, the optimum configuration with the maximum VS is found to start the barrier sequence by a robust Ge_4 sub-layer.

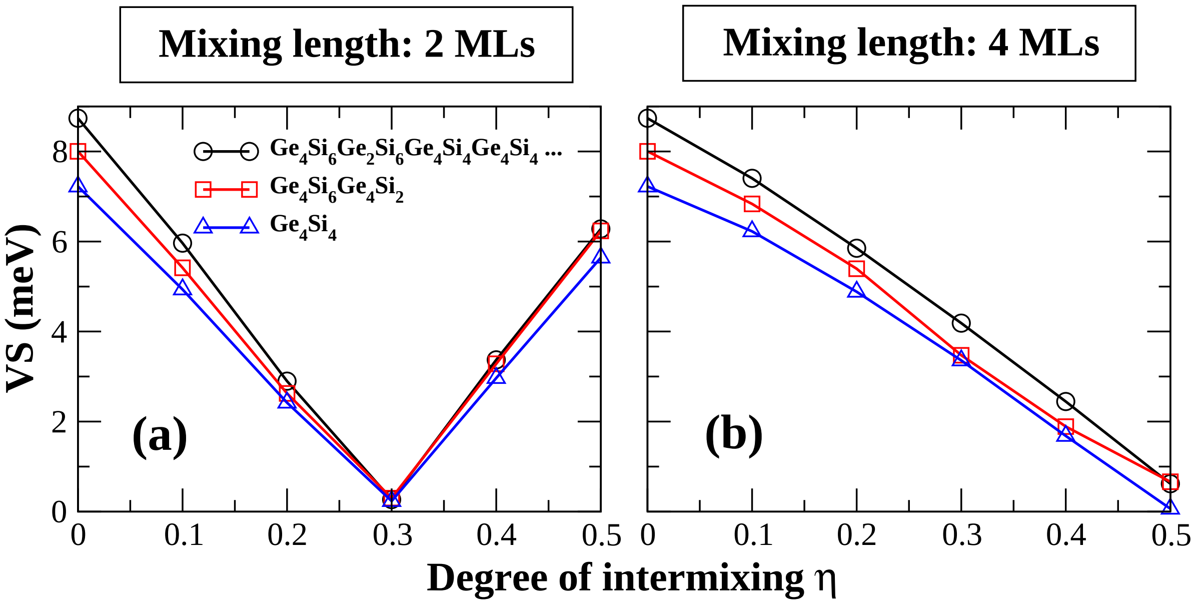

Supplementary Figure S3: **Effect of atomic inter-diffusion on VS for more ordered barriers.** Calculated VS (in meV) for a 40 MLs Si quantum well embedded in several ordered superlattice barriers on a 40% Ge substrate, as a function of the degree of inter-layer mixing, η, for different mixing length of 2ML (a) and 4ML (b). We considered three superlattice barriers: the best configuration for the total thickness of 80 MLs ($Ge_4Si_6Ge_2Si_6Ge_4Si_4Ge_4Si_4 \ldots$), the best configuration for the shorter period of 16 MLs ($Ge_4Si_6Ge_4Si_2$), the best configuration for the simplest Ge_nSi_n superlattice (Ge_4Si_4). At $\eta = 0$ there is no mixing and $\eta = 0.5$ means the maximum diffusion, i.e. complete destruction of Si-rich or Ge-rich pattern layer. The mixing length defines the maximum range at the interface where the mixing occurs.

To further explore the effect of atomic inter-diffusion on VS for ordered superlattice barriers, we performed more calculations similar to that of Fig. 4b in the main text for other superlattice barriers. Supplementary Figure S3 shows the calculated VS as a function of degree of inter-layer mixing, η for the best configuration of the 80 MLs thickness (shown in Fig. 4b in the main text), the shorter period of 16 MLs, and the simplest Ge_nSi_n superlattice barriers on %40 Ge substrate when two cases of mixing lengths [2 MLs (a) and 4 MLs (b)] are considered. We find all the superlattice barriers

exhibit the consistent behavior as addressed in the main text. In particular, for both mixing lengths, the enhanced VS in ordered superlattice barriers is preserved with the reasonable inter-layer mixing ($\eta < 0.1$). For the shorter mixing length (2 MLs), the VS is initially suppressed more dramatically by the inter-layer mixing, and is recovered to a relatively high value when the larger mixing occurs.

Supplementary Note 1

Origin of oscillation of VS *vs* odd/even well thickness. As shown in Fig. 3b of the main text, while the VS of effective mass approximation (EMA) with continuum d_{con} show a much faster oscillation, they reproduce well the existence of independent oscillation for discrete d of odd and even MLs. This clearly demonstrates the underlying origin of such even-odd independent oscillations. In particular, the dependence of EMA VS on continuum d_{con} (solid black line in Fig. 3b of the main text) oscillates as a rectified sinusoid wave with a wavelength $\lambda_{VS} = 2\pi/(2k_0) \sim 2.4$ MLs, where $k_0 = 0.85(2\pi/a_0)$ is the wavevector at the conduction band minimum along the Δ $(\Gamma - X)$ direction and a_0 is the lattice constant of Si. The probing period of the pseudopotential (PP) data, i.e. the physically accessible sampling rate for thickness d is $t_{PP} = 1\,\mathrm{ML}$, is incommensurate with the oscillation of VS with d_{con}. The maximum (Nyquist) wavelength that can capture the fast oscillation of VS with continuum d_{con} (in principle smaller than $\lambda_{VS}/2$) is further reduced by the fact that the data is taken as an absolute value, which has Fourier components with higher wave-vector compared to the simple sinusoid. This particular t_{PP}/λ_{VS} ratio generates apparent even-odd independent oscillations.

Although the EMA offers a qualitative explanation for this effect, the quantitative selection of an appropriate value of thickness d is in principle not reliably described within EMA because of the unavoidable ambiguity on the interface positioning relative to the realistic atomic planes, so the location of the barrier potential could be anywhere in the interfacial monolayer region and the uncertainty for d would be larger than 1ML. As we will show in the following Supplementary Note 3, the multiple scatterings between layers in a superlattice barrier can be engineered to give constructive interference, being the underlying foundation of the remarkably enhanced VS we achieve in this work.

Supplementary Note 2

Possibility of forming strain-induced dislocation defects. A high epitaxial strain (*i.e.* large Ge content in the substrate relative to the Ge-poor film to be grown) is

not an essential condition for significant enhancement of valley splitting. This is reflected, for example, by the fact that the optimal configuration of the ordered superlattice barrier with no Ge in the substrate (Fig. 2d in the main text) is predicted to give a VS magnitude of ∼6 meV, and that the optimal configuration on a substrate containing 20% Ge content (Fig. 2e) is predicted to have a VS above 7 meV (as listed in Table I in the main text). Although these values are somehow lower than that of the optimum configuration on the 40% Ge substrate (8.7 meV), the enhancement is still remarkable by comparison with the values for alloy barriers. Nevertheless, some of our structures are coherently strained, we discuss here the possibility of formation of dislocation defects (that are widely observed in strained epitaxial film growths) and their potential effect on the VS.

(a) Likelihood of formation of dislocations in some of our predicted optimal structures. Dislocation-free coherently strained epitaxial $Si_{(1-x)}Ge_x$ film can be grown on either bulk silicon substrate or on relaxed Si-Ge buffer layer on top of Si substrate if the film thickness does not exceed the critical thickness H_c(x). Beyond this thickness, misfit-induced strain (that can not be accommodated elastically) leads to the formation of dislocation defects or islands on the surface [8, 41]. Our proposed structures for optimum VS consist of a film made of pure Si quantum well (40 MLs thick) surrounded on each side by 80 MLs of Si-Ge layer-by-layer superlattice barrier (see Fig. 1d in the main text) with Ge content between 40% and 50% in the barrier (see Figs 2d-f in the main text). The designed films are to be grown on Si-Ge alloy substrates with Ge content between 0 and 40%.

(i) For the 0% Ge substrate (*i.e.* bulk silicon wafer), the Si well within the film can be readily grown in quite thick layers, but the growth of Si-Ge barrier on top of the substrate is limited by the misfit (between it and the substrate) of 1.5-2.0%, which gives a Matthews and Blakeslee equilibrium critical thickness H_c(barrier) of 4-6 nm [46]. Under non-equilibrium condition the formation of metastable layers increases H_c(barrier) to 12-15 nm [47, 48]. The thickness of the barrier considered in our calculations is 80 MLs (10-12 nm), which is compatible with H_c(barrier).

(ii) As for the 40% Ge (relaxed virtual) substrate, where the maximum VS (8.7 eV, see Table I in the main text) is achieved, because of the small misfit (0-0.4%) between barrier and substrate, the growth of a thick barrier on the lower side of Si well is not a problem. Then, the growth of Si well on top of the barrier will be limited by the Matthews and Blakeslee equilibrium H_c(well) of 4-6 nm, which is comparable to the well thickness we considered, 40 MLs (5-6 nm). Once the Si well is grown without dislocations, the continuous growth of another upper barrier is possible.

On the other hand, note that the critical thickness H_c(x) can be significantly increased

in the strained epitaxial growth by a reduction in growth area [49, 50]. Meanwhile, it is reasonable to expect that the integrity of the Si/Ge superlattice barrier close to Si well will play an important role in controlling the VS. As such, the thickness of barriers might be to some extent reduced without losing optimum VS. Moreover, in experimental sample preparation for Si qubits usually one barrier is grown on one side of Si well, and a confining electric field is applied from the other side [6].

Therefore, these analyses suggest that obtaining dislocation-free Si/barrier films, following our proposed optimal structures of barriers and grown over 0-40% Ge substrates is quite promising.

(b) Effect of dislocations on VS. In case that a minor amount of dislocation defects is formed in experimental growth, two main effects are expected to be induced: the first is the possible emergence of mid-gap defect states caused by the dislocation core, which is known to be detrimental to electronic and optical applications of semiconductors; the second is the long-range elastic strain field in the region away from the dislocation core [51]. It is not trivial to explicitly simulate dislocations with atomistic scale calculations because of the structural complexity of various dislocations. Here we probed the effect of the elastic strain field induced by dislocations in thick Si well region by introducing a line of Ge atoms replacing a line of Si atoms (to mimic the dislocation core). We simulate both misfit and threading dislocations by substituting Si atoms along the [100] direction (lying in Si/barrier interface) and the [001] direction (across Si well), respectively. These directions are not necessarily the natural directions of actual dislocations (misfits are oriented along the [110] direction typically, for instance), but it should give an idea of the effect of defects along and across the Si/barrier heterostructure, respectively. We expect that, to some extent, such calculations may capture basic features of the long-range strain field induced by the dislocation core. The calculated VS is $\sim$8.8 and $\sim$8.7 meV for the structure with the mimicked misfit and threading dislocation, respectively, which show a negligible change in comparison with the value (8.7 meV) of the dislocation-free structure. From such rather rough calculations, it is conjectured that even if a moderate dislocation density appears in the thick-layer growth, the effect of their induced elastic strain field on the VS magnitude may not be notable.

(c) General remark about structural defects. Finally we point out that the negative effect of structural defects in the Si active layer or at the Si/barrier interface is that the complicated electronic states induced by the defects may be difficult to be explicitly predicted. The defect formations break the underlying atomic arrangement and original coherence, and also constitute pinning or repulsive regions for the electron. Therefore

electronic spin control, for example tuning exchange coupling between electron spin pairs, becomes a harder issue, even if the VS may not be changed dramatically. Coherent growth is likely to take place on the relatively moderate 20% Ge substrate, which constitutes a more conservative choice for initial experimental trials. As mentioned above, the optimal barrier structure we predicted on this substrate provides the VS above 7 meV, still much larger than the values attainable for alloy barriers and adequate in defining spin-only qubits.

Supplementary Note 3

Origin of remarkable VS enhancement in the Si/Ge_4 motif. From the results of inverse-band-structure search, we find that the ordered layer-by-layer stacking of superlattice barriers can be exploited to reach a remarkably enhanced VS. Moreover, a "magic" motif is identified for all the optimum configurations: the first Ge sub-layer is always 4 MLs thick. We tackle the problem of what is special about this particular thickness using the effective mass approximation (EMA) approach.

A quantum well of thickness $2L$ cladded by Ge slabs of thicknesses L_B from both sides can be analyzed within the EMA as a set of four Heaviside step functions of barrier height U_0. The first pair is located at the positions $z = +L$ ($z = -L$), with the potential ascending as we move towards higher (lower) z, forming the well. The second pair is located at $z = \pm(L + L_B)$, with the profiles opposing the first pair, *i.e.*, descending at $z = L+L_B$ and ascending at $z = -L-L_B$. This way, the second pair forms an "anti-well".

From the theory shown in Ref. 23, the VS is determined by the electronic wavefunction at the interface and the spatial derivative of the electrostatic potential under certain approximations. Taking the step function model, the above inner pair will contribute to VS as a pair of δ-functions. The anti-well formed by the above outer pair will have a VS that has opposite sign when compared to a regular well of thickness $2L + 2L_B$.

By analyzing Fig. 3a in the main text, we can check what values of L_B would lead to constructive or destructive interference. For instance, we analyze $L_B = 2$ MLs by picking a quantum well thickness (we chose $2L = 40$ MLs for the calculations in the main text) and comparing it to a quantum well of thickness 4 MLs larger (44 MLs here). We readily obtain that they are both near oscillation peaks with same sign (the sign may be inferred by carefully counting the oscillation nodes between the two data points). Therefore, the anti-well of 44 MLs induces a strong destructive interference on the VS generated by the 40 MLs quantum well. This explains the suppressed VS for the Si_2Ge_2 superlattice barrier

in Fig. 5a of the main text. The same argument clarifies the enhancement at $L_B = 4$ MLs (*i.e.*, the Si_4Ge_4 superlattice barrier), that is, the constructive interference between a 40 MLs thick well and a 48 MLs thick "anti-well".

Supplementary Methods

A. Structures for VS optimization. The [001]-oriented Si quantum well and cladding Si-Ge based barriers are constructed from the diamond structure. The length scale in this work is in the unit of monolayers (MLs), where 1 ML is equal to $1/4a_0$ (a_0 is the cubic lattice constant). For the well embedded in ordered superlattice barriers, the barriers on both sides are made symmetric to the center of the well, i.e, if the center of the well is at $z = 0$, the layers at $z = l$ and $-l$ are occupied by the same species. Periodic boundary conditions are adopted, with barrier thickness of 80 MLs on both sides of the well, which has been checked to ensure the calculated VS converged within 0.1 meV, i.e. enough to isolate the well from its images interacting. To mimic random alloy barriers, we construct the 8x8 in-plane supercells perpendicular to the z direction, and randomly mix Si/Ge atoms for site occupations in terms of specific compositions. We employ a virtual epitaxial substrate with the in-plane lattice constant interpolated in terms of Si-Ge alloy composition, to which the whole system is coherently strained. Since too high Ge content in substrate is known to cause dislocations in thick active Si layers (to relieve excessive strain), we consider the substrates with the Ge content up to 40%. The strain energy is minimized with a generalized valence force field functional parametrized for diamond Si/Ge by fitting the results of density functional theory (DFT) [45], without dislocation or other defects allowed.

B. Atomistic empirical pseudopotential calculations. The energies and wavefunctions of conduction valley states were calculated by using the atomistic pseudopotential method, as described in detail in Refs. 24 and 25. Briefly, we numerically solve the Schrödinger equation of the quantum well system

$$[-\frac{\hbar^2}{2m}\bigtriangledown^2 + \sum_{n,\alpha} v_\alpha(\mathbf{r} - R_{n,\alpha}) + \sum_\alpha V_\alpha^{SO}]\varphi_i(\mathbf{r}) = \epsilon_i\varphi_i(\mathbf{r}), \quad \text{(Supplementary Equation S1)}$$

where the Hamiltonian consists of the kinetic-energy (first term), atomistic screened pseudopotential (second term) and spin-orbit coupling (third term). The screened pseudopotential is fitted to accurately reproduce electronic properties of bulk Si and Ge (e.g. interband transition energies, effective masses, spin-orbit splittings, deformation potentials), as

well as combined Si/Ge systems such as interface band-offsets, and random alloy bowing parameter. This atomistic Hamiltonian (overcoming the DFT limitations on electronic structure calculations), accompanied with a plane-wave basis set and folded-spectrum diagonalization [52], allow us to accurately calculate the VS value at the order of meV or lower for numerous candidate structures with economic efficiency. The band offset of valley states between Si well and barrier is evaluated directly by the energy difference between the conduction valleys of Si and barrier, which have been folded onto the zone center by using large enough supercells along the Z-direction.

C. Effective mass calculations. We essentially adopt the model detailed in Ref. 23. The approach is further simplified by taking (i) couplings between plane waves with $\mathbf{G} + \mathbf{G}' = 0$ only; (ii) the approximation of deep, thick quantum wells, so that the wavefunction penetration in the barrier does not depend on the ground state energy, as described in Ref. 19. It should be pointed out that the qualitative results obtained do not depend on these approximations. In order to describe parity flips in the wave-function as the quantum well thickness is increased, the evanescent wavefunction contribution must be included. We do so by limiting the results to the first order in κ/k_0, where κ is the exponential damping factor of the wavefunction inside the barrier (inverse of the skin depth). Within these assumptions, the VS is a purely real number.

D. Inverse-band-structure approach. To deal with an astronomical number of candidate layer-stacking configurations for ordered superlattice barriers, we employ the developed inverse-band-structure approach [26, 27, 35, 36], i.e., a biologically inspired genetic algorithm (GA) to guide the atomistic pseudopotential calculations, searching for the optimum configuration of superlattice barriers that gives the maximum VS. Particularly this is done based on the Darwinian evolution rules of survival of the fittest. The initial population of configurations ("individuals") is generated randomly. In each evolution step ("generation") individuals with good target property ("fitness") survive, a percent of individuals with bad fitness are abandoned, and at the same time new individuals are generated by defined GA operators. In the present work, the fitness is the energy splitting between two lowest valley states of the Si quantum well. To avoid occasional evolution, in each case of GA calculation the results are collected among four different trials.

Supplementary References

[46] Matthews, J. & Blakeslee, A. Defects in epitaxial multilayers: I. misfit dislocations. *Journal of Crystal Growth* **27**, 118–125 (1974).

[47] Bean, J., Feldman, L., Fiory, A., Nakahara, S. & Robinson, I. Ge_xSi_{1-x}/Si strained-layer superlattice grown by molecular beam epitaxy. *Journal of Vacuum Science & Technology A* **2**, 436–440 (1984).

[48] People, R. & Bean, J. Calculation of critical layer thickness versus lattice mismatch for GeSi/Si strained-layer heterostructures. *Applied Physics Letters* **47**, 322 (1985).

[49] Fitzgerald, E. A. *et al.* Elimination of interface defects in mismatched epilayers by a reduction in growth area. *Applied Physics Letters* **52**, 1496–1498 (1988).

[50] Kumar, A. & Subramaniam, A. Position dependant critical thickness in finite epitaxial systems. *Applied Surface Science* **275**, 60–64 (2013).

[51] Hirth, J. P. & Lothe, J. Theory of dislocations. In *Theory of dislocations* (John Wiley and Sons, Inc., Boston, MA, 1982).

[52] Wang, L. & Zunger, A. Solving schrödinger's equation around a desired energy: Application to silicon quantum dots. *The Journal of Chemical Physics* **100**, 2394–2397 (1994).
